# An Investigation on a Crystalline-Silicon Solar Cell with Black Silicon Layer at the Rear

**DOI:** 10.1186/s11671-017-2388-y

**Published:** 2017-12-15

**Authors:** Zhi-Quan Zhou, Fei Hu, Wen-Jie Zhou, Hong-Yan Chen, Lei Ma, Chi Zhang, Ming Lu

**Affiliations:** 1 Department of Optical Science and Engineering and Shanghai Ultra-Precision Optical Manufacturing Engineering Center, Fudan University, Shanghai, 200433 China; 2 Department of Micro-electronics, Fudan University, Shanghai, 200433 China

**Keywords:** c-Si solar cell, Black silicon, Graded band gap, Surface recombination

## Abstract

Crystalline-Si (c-Si) solar cell with black Si (b-Si) layer at the rear was studied in order to develop c-Si solar cell with sub-band gap photovoltaic response. The b-Si was made by chemical etching. The c-Si solar cell with b-Si at the rear was found to perform far better than that of similar structure but with no b-Si at the rear, with the efficiency being increased relatively by 27.7%. This finding was interesting as b-Si had a large specific surface area, which could cause high surface recombination and degradation of solar cell performance. A graded band gap was found to form at the rear of the c-Si solar cell with b-Si layer at the rear. This graded band gap tended to expel free electrons away from the rear, thus reducing the probability of electron-hole recombination at b-Si and improving the performance of c-Si solar cell.

## Background

Highly surface-etched Si that has been loaded or doped with metal or non-metal ions could exhibit strong and broadband absorptivity [1–6]. This type of Si, or black Si (b-Si), has attracted much attention for its potential application in broadband response photovoltaics [7–9]. To date, investigations of b-Si solar cell have focused on such a configuration that the b-Si layer is at the front of the solar cell [10–19]. In this case, electron-hole pairs induced by the sub-band gap near infrared (NIR) absorption at the b-Si layer are far away from the PN junction zone and cannot be decomposed by the built-in field to become charge carriers, making the sub-band gap NIR photovoltaic response impossible. It is then conceived that if the b-Si layer is placed at the rear, the NIR absorption-induced electron-hole pairs could be decomposed by the Si/oxide interfacial field at the rear [20] or by a built-in field there if an interdigitated back contact (IBC) configuration is adopted [21], making the photovoltaic (PV) response of such a crystalline (c)-Si solar cell extend to the sub-band gap NIR range. Unfortunately, the large specific surface area of b-Si would usually cause high surface recombination, which would severely degrade the solar cell performance [10, 15, 22]. Hence, before we start to study the sub-band gap NIR response of c-Si solar cell, it is necessary to know how large the surface recombination of b-Si could be and how to minimize or avoid its influence [23]. In this work, we studied the PV response of c-Si solar cell with b-Si at the rear and explored the physics underlying our observations.

## Methods

### Materials

P-type Si<100> wafer (CZ, double-side polish, 10 × 10 × 0.2 mm^3^ in size, 1–10 Ω cm) was used as the substrate. The Si wafer was ultrasonically cleaned and then dipped in dilute HF(1%), followed by etching in a NaOH/alcohol/H_2_O (0.5 g/200 ml/200 ml) solution at 90 °C for 15 min to slightly texture the surface for antireflection and then rinsing in de-ionized water. To prepare b-Si at the rear, a Ag layer with apparent thickness of 3 nm was evaporated onto one surface of Si substrate as catalyst by resistance heating in a home-made vacuum chamber with base pressure less than 5 × 10^−4^ Pa. After immersing the Si wafer in a HF(40%):H_2_O_2_(30%):H_2_O = 1:5:10 solution for 120 s at room temperature, a b-Si layer was formed at that Si surface or at the rear of the solar cell. A phosphorous paste was then deposited onto the other Si surface or the front of the solar cell, followed by annealing at 900 °C for 20 min in nitrogen to form a PN junction. A 20-nm-thick SiO_2_ layer was evaporated onto the front of the solar cell for surface passivation. For the rear surface passivation, a 10-nm-thick Al_2_O_3_ layer was deposited using the technique of atomic layer deposition (ALD) (Beneq TFS 200). An 80-nm-thick ITO layer was deposited onto the front surface as the front electrode. A 2-μm-thick Al layer was evaporated by resistance heating as the rear electrode. A thermal annealing in nitrogen at 425 °C for 5 min was conducted to finalize the preparation of c-Si solar cell. It should be pointed out that in this work, we focused on the effect of b-Si at the rear on the PV response; therefore, the front surface was only slightly textured and not highly etched to form b-Si.

### Measurements

The reflectance spectra were measured using a UV-vis-NIR spectrophotometer (Shimadzu, UV-3101PC). The surface morphology was measured with a scanning electron microscope (SEM) (Philips, XL 30). The PV parameters of the solar cell were obtained with a solar simulator (Oriel/Newport, model 94023A) under 1-Sun AM1.5G condition. The external quantum efficiency (EQE) of the solar cell was acquired on a QE system of Oriel/Newport. Transmission electron microscopy (TEM) measurements were carried out on a JEOL EM-3000 system. Surface-emitting photoluminescence (PL) spectra were recorded by a spectrophotometer (Ocean Optics USB2000), with a 325-nm He-Cd laser (Melles Griot, model series 74) as the excitation source. The surface potentials of p-type Si and b-Si were measured by a Kelvin probe system (KP Technology SKP5050), the so-called contact potential difference, or CPD identification.

## Results and Discussion

**Fig. 1:**
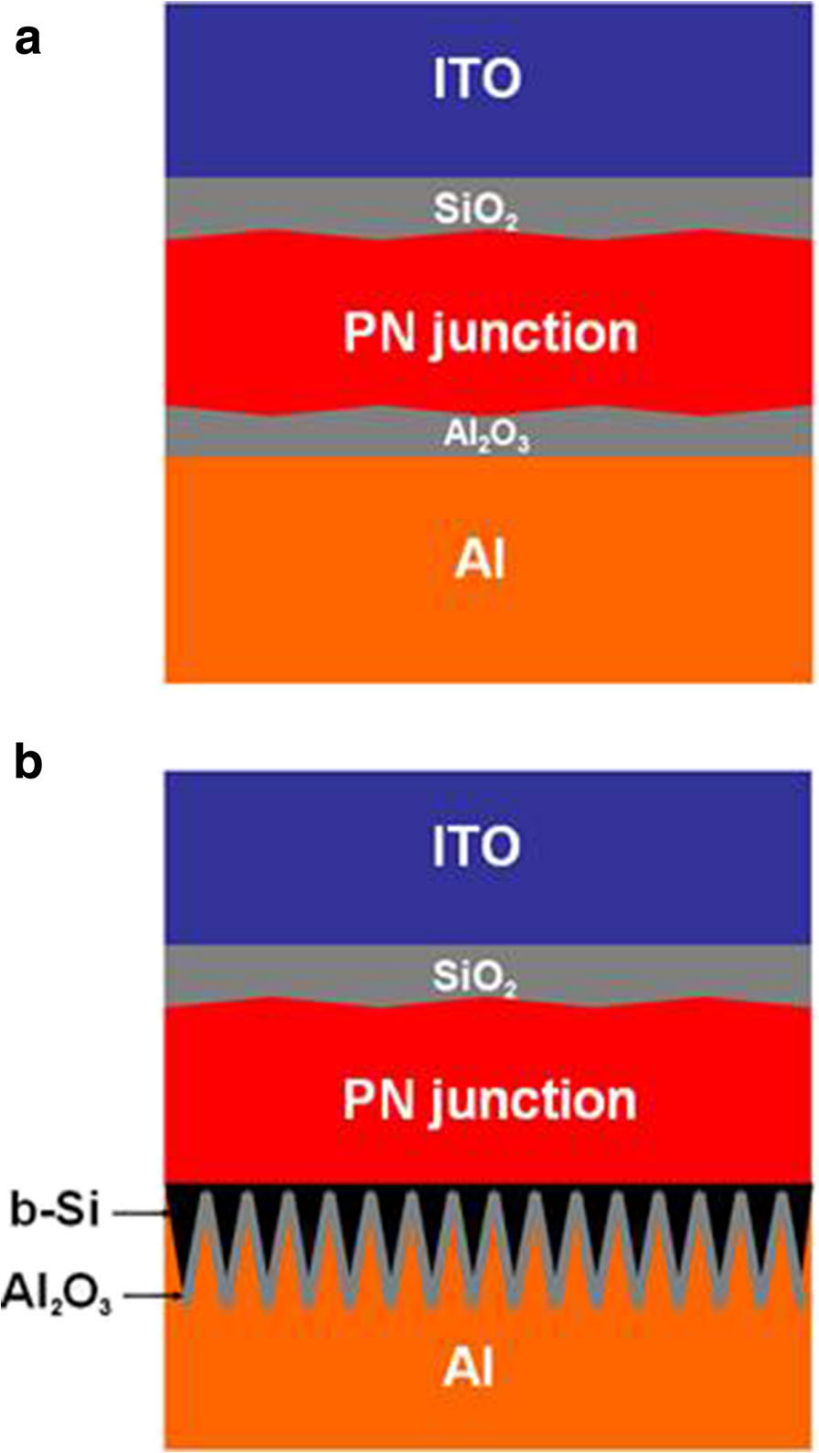
Schematics of slightly surface-textured c-Si solar cells without (**a**) and with (**b**) b-Si at the rear

Figure 1a shows a schematic of a slightly surface-textured c-Si solar cell after front and rear passivations. Figure 1b gives a schematic of a similarly structured solar cell but with b-Si at the rear. The thickness of the solar cell is approximately 200 μm.

**Fig. 2:**
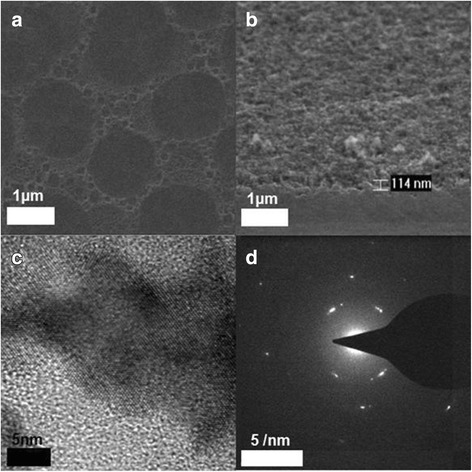
SEM images of surface-textured Si (**a**) and surface-etched b-Si (**b**), HRTEM (**c**), and SAED (**d**) of b-Si

Figure 2a shows a top-view SEM image of the textured front surface. Figure 2b gives a side-view SEM image of the b-Si surface. The average height of nanostructure of textured Si is 10~20 nm, while that of b-Si is ~ 110 nm. Figure 2c shows a high-resolution (HR) TEM image of b-Si, where the nanocrystalline Si is discernible as reflected by the diffraction fringes. This crystallinity of b-Si is also indicated by the SAED (selected area electron diffraction) pattern as shown in Fig. 2d.

**Fig. 3:**
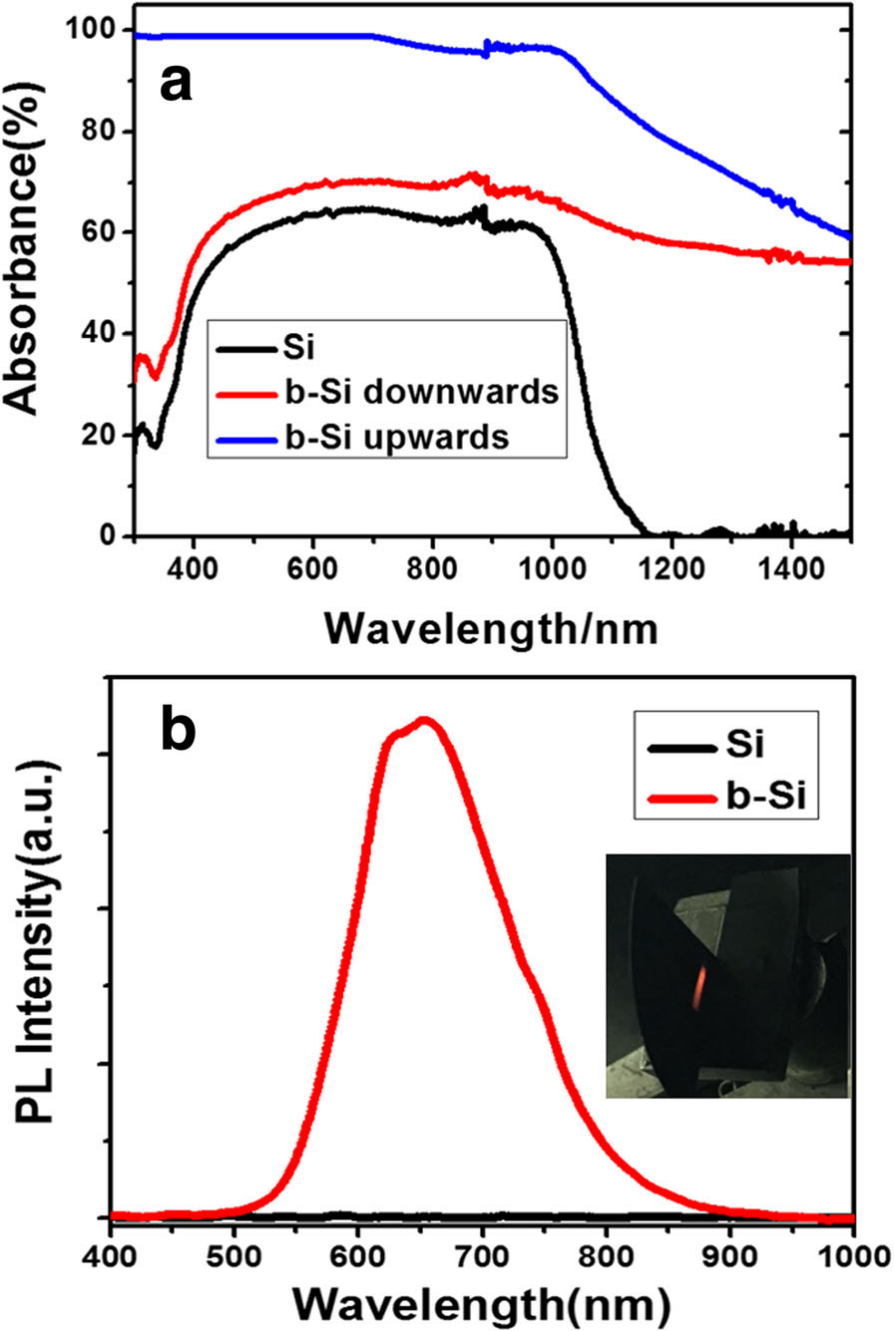
Absorption spectra of a wafer Si, b-Si that faces the incident light, and b-Si with its back toward the incident light (**a**). PL of Si and b-Si under illumination of a 325-nm exciting laser (**b**). The inset shows the b-Si under illumination of the 325-nm laser

Figure 3a gives the absorption spectra for a wafer Si (termed “Si”), b-Si that faces the incident light (termed “b-Si upwards”), and b-Si with its back toward the incident light (termed “b-Si downwards”). For “Si,” it is seen that when the photon energy is less than the c-Si band gap width (1.1 eV), or equivalently, the wavelength is larger than 1100 nm and almost no absorption occurs as expected. However, for “b-Si upwards,” in addition to the large enhancement of absorption in the 300–1100-nm range due to the strong light trapping by the nanostructures of b-Si [1–9, 24–31], the sub-band gap NIR absorption appears. This sub-band gap absorption could be attributed to the formation of impurity levels within the band gap, which allows absorption of lower energy photons [25–28, 32]. The sub-band gap absorption can be efficient with the assistance of light trapping [25–28, 32]. For “b-Si downwards,” the absorption in the 300–1100-nm range increases as compared to that of “Si.” It was noticed that although there was no Ag deposited on this front side, it would still be slightly textured during the formation of b-Si at the rear. This surface texturing strengthened the light trapping. It is seen that although part of the sub-band gap NIR is reflected at the front surface, most of the NIR absorbance still remained. This is what one needs for developing a sub-band gap NIR response c-Si solar cell in the future. Figure 3b gives a measured PL spectrum of b-Si, and the inset figure is a photograph of the b-Si under the illumination of the 325-nm laser. No PL emission is found for the Si wafer. The PL emission from b-Si is another indication that Si nanocrystals exist as shown in Fig. 2c [10, 33].

**Fig. 4:**
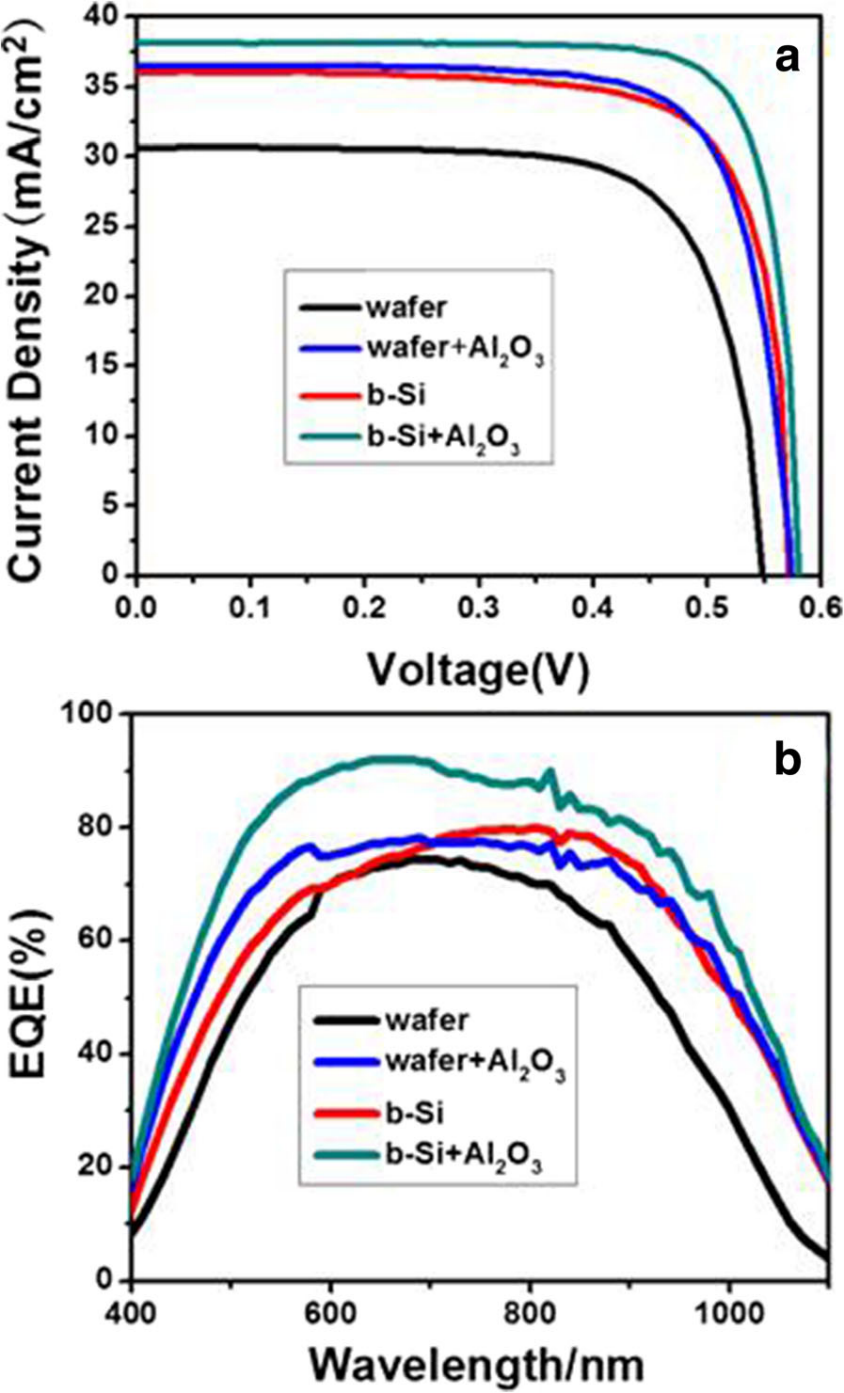
Photovoltaic *J*-*V* (**a**) and EQE curves (**b**) for the solar cells of “wafer,” “wafer + Al_2_O_3_,” “b-Si,” and “b-Si+Al_2_O_3_”

We now investigate how the b-Si at the rear of the c-Si solar cell would affect its performance. In the following, the b-Si solar cell means the c-Si solar cell with a b-Si layer at the rear. For comparison, we have made four c-Si solar cells, i.e., a wafer Si solar cell (termed “wafer”), wafer Si solar cell with Al_2_O_3_ passivation at the rear (termed “wafer + Al_2_O_3_”), b-Si solar cell (termed “b-Si”), and b-Si solar cell with Al_2_O_3_ passivation at the rear (termed “b-Si + Al_2_O_3_”). All the four solar cells have been textured at the front surface. The current density-voltage (*J*-*V*) curves of the four solar cells are shown in Fig. 4a, and their EQE curves are shown in Fig. 4b. The corresponding PV parameters including open-circuit voltage (*V*_OC_), short-circuit current density (*J*_SC_), fill factor (FF), and photoelectric conversion efficiency (*η*) are given in Table 1. As compared to the “wafer Si” solar cell, after rear passivation by Al_2_O_3_, the cell of “wafer + Al_2_O_3_” shows a far better performance. The *J*_SC_, *V*_OC_, FF, and *η* are increased, and a considerable enhancement of EQE is seen in the whole measured wavelength range. This result is consistent with the previous reports as the surface recombination has been well suppressed by Al_2_O_3_ passivation [34–36]. When the b-Si layer exists at the rear, significant decreases in *J*_SC_, *V*_OC_, and *η* of the “b-Si” cell would be expected because of the high surface recombination due to the large specific surface area of b-Si, as compared to the “wafer” cell [15, 22]. However, on the contrary, the performance of “b-Si” turns out to be much improved, with its efficiency even close to that of “wafer + Al_2_O_3_,” and having a relative 27.7% increase. The EQE curve also shows a considerable broadband enhancement. Large surface area-induced high surface recombination seems not to happen here. We then go on to check the cell of “b-Si + Al_2_O_3_” and find that after Al_2_O_3_ passivation at the rear, *J*_SC_, *V*_OC_, FF, and *η* further increase and so do the EQEs. This indicates that Al_2_O_3_ still passivates efficiently the rear surface as in the case of “wafer + Al_2_O_3_.” The role played by b-Si at the rear is unexpectedly interesting and needs to be further explored.

**Table 1:** Photovoltaic parameters for the solar cells of “wafer,” “wafer + Al_2_O_3_,” “b-Si,” and “b-Si + Al_2_O_3_”

Sample	*V*_oc_ (mV)	*J*_sc_ (mA/cm^2^)	FF (%)	*η* (%)
Wafer	548	30.64	73.33	12.33
Wafer + Al_2_O_3_	574	36.49	75.66	15.84
b-Si	571	36.02	76.48	15.75
b-Si + Al_2_O_3_	581	38.11	81.02	17.96

**Fig. 5:**
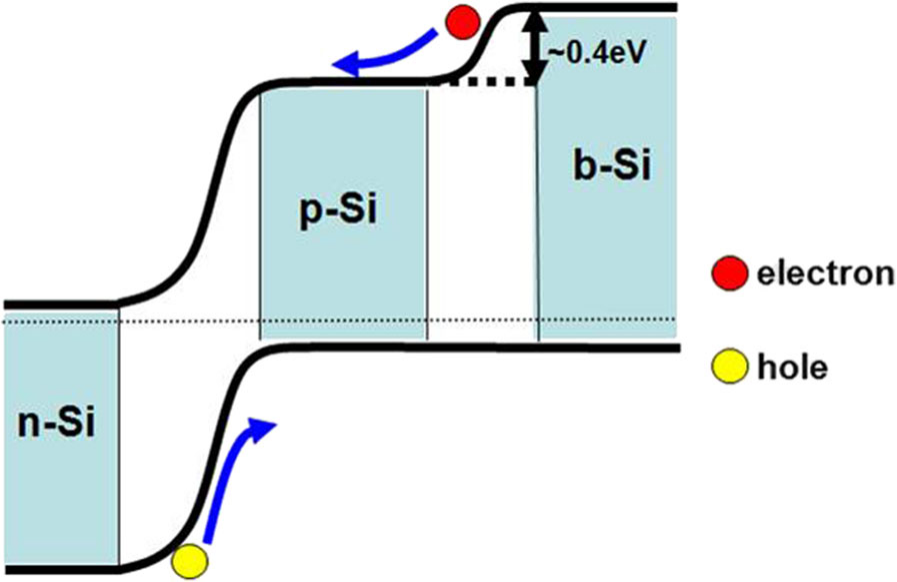
Energy band diagram of the PN junction with b-Si at the rear

Figure 5 shows an energy band diagram of the PN junction with b-Si at the rear. That the conduction band minimum of b-Si is 0.4 eV above that of p-type Si results from the CPD measurement. Since the b-Si is directly grown on the very p-type Si, the distance between the Fermi energy level and the valence band maximum should basically be kept the same as the doping concentration is the same [37]. Therefore, the band gap width of b-Si is larger than that of wafer Si. This is consistent with the formation of nanocrystalline Si, their PL emission as shown in Figs. 2c and 3b, respectively, and the quantum confinement effect [38]. With such a graded band gap at the rear, free electrons would be expelled away from the b-Si and rear electrode [39]; meanwhile, the drifting of holes toward the rear electrode is not affected, as indicated in Fig. 5. In that manner, the probability of electron-hole recombination at b-Si can be largely reduced and the problem of high surface recombination be efficiently avoided. The formed graded band gap explains why the “b-Si” cell has a far better performance than the “wafer” cell, even though its specific surface area is much larger.

**Fig. 6:**
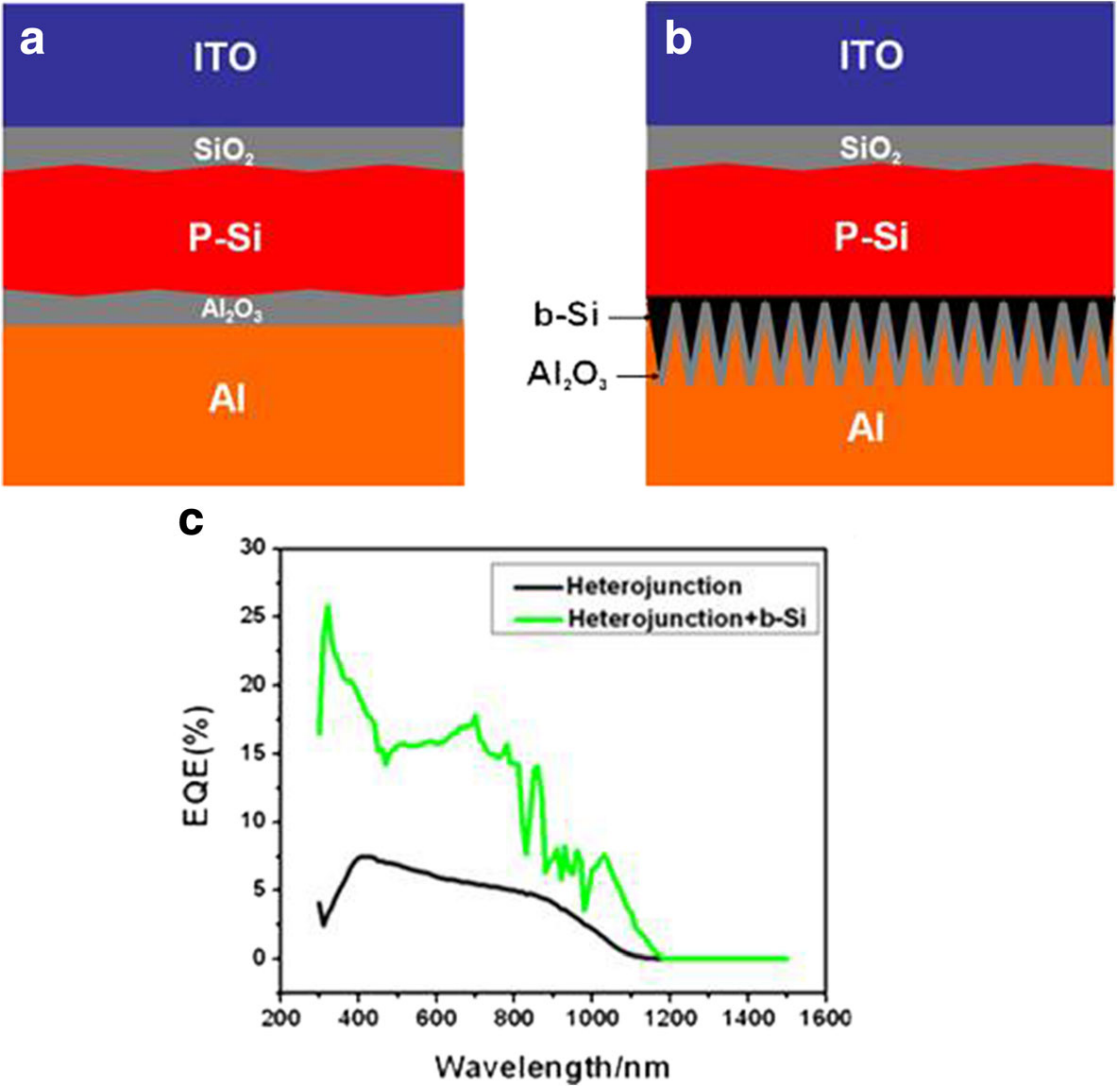
Schematics of a heterojunction structured PV device without (**a**) and with (**b**) b-Si at the rear and their EQE curves (**c**)

The positive role of b-Si at the rear in photovoltaics was further manifested in heterojunction structured PV devices as indicated in Fig. 6a, b. As shown in Fig. 6c, for this PV device with b-Si at the rear, the EQE was obviously enhanced as compared to that with no b-Si at the rear. The graded band gap at the interface of P-Si and b-Si should be responsible for the enhancement of EQE [39, 40]. This result is consistent qualitatively with that in Fig. 4b. Although the PV configurations for Fig. 4b and Fig. 6c are different, the role played by the b-Si at the rear is basically the same.

## Conclusions

We studied the c-Si solar cell with a b-Si layer at the rear. The c-Si solar cell of such a configuration showed a far better performance than a c-Si solar cell of similar structure but with no b-Si at the rear. This result was attributed to the formation of a graded band gap at the rear, which can largely reduce the probability of surface recombination at the rear, thus improving the performance of the c-Si solar cell. The finding of this work can be applied to developing a c-Si solar cell with broadband PV response, including the sub-band gap NIR response, in the future.
